# Efficacy and Complications Associated with Acellular Dermal Substitute Use in the Treatment of Acute Burns: A Systematic Review and Meta-Analysis

**DOI:** 10.3390/ebj4040036

**Published:** 2023-10-23

**Authors:** Isobel Press, Naiem Moiemen, Zubair Ahmed

**Affiliations:** 1 College of Medical and Dental Science, University of Birmingham, Edgbaston, Birmingham B15 2TT, UK; 2 Institute of Inflammation and Ageing, University of Birmingham, Edgbaston, Birmingham B15 2TT, UK; 3 The Scar Free Centre for Conflict Wound Research, Queen Elizabeth Hospital, University Hospitals Birmingham Foundation Trust, Mindelsohn Way, Birmingham B15 2TT, UK; 4 Centre for Trauma Sciences Research, University of Birmingham, Edgbaston, Birmingham B15 2TT, UK

**Keywords:** burn, skin substitute, artificial dermis, dermal substitute, dermal regeneration template

## Abstract

Over several decades, skin substitutes have become an essential tool in acute burn surgery, particularly in major burns, where scarce donor tissues can limit the availability of autografts. This systematic review aimed to assess the efficacy, complication rates, and long-term outcomes of acellular dermal substitutes in acute burns and compare these to conventional skin grafting methods of coverage. A search of PubMed, Web of Science, and CENTRAL for appropriate randomized controlled trials (RCTs), non-randomized trials, and observational studies was conducted. Following screening, nine RCTs and seven observational studies fulfilled our inclusion and exclusion criteria. Our primary outcomes, which were graft take and incidence of infection, found no significant difference between the substitute and control procedures in a meta-analysis (*p* = 0.37 and *p* = 0.87, respectively). For our secondary outcomes, the studies were analyzed via narrative synthesis, which reported variable rates of graft loss and duration of acute hospital stay, from which definitive conclusions could not be drawn due to the heterogeneity in reporting. Despite a high risk of bias in the included studies, the evidence reviewed suggests that the treatment of an acute burn with a substitute may improve scar quality when compared to conventional grafting. This review therefore suggests that acellular dermal substitutes offer a viable method for staging the closure of deep partial- and full-thickness acute burns, although more robust RCTs with less heterogeneity are needed to support these conclusions.

## 1. Introduction

Since the development of the Integra^®^ dermal regeneration template (Integra^®^ Life Science Corporation, Plainsboro, NJ, USA) in 1981 by Yannas and Burke [1], skin substitutes have become firmly established in the field of burn care as an effective method for the coverage of acute burns, following early excision. Skin substitutes aim to mimic the innate qualities of human skin, restoring anatomy and physiology, providing protection from infection, and accelerating healing [2]. They therefore provide a suitable method to aid wound closure in deep partial- and full-thickness burns when delayed closure is warranted due to limited donor split-thickness skin grafts (STSGs), the current gold standard for acute burn coverage [3]. However, coverage of full-thickness burns via this traditional STSG alone is often not ideal, resulting in tissue fragility, poor scar quality, and abnormal sensation due to incomplete dermal regeneration, which substitutes aim to counteract by providing a more favorable wound bed for later STSG application [4,5]. Substitutes are also of particular value in extensive burns, in which there are limited autograft sites available, and when there is concern over producing multiple iatrogenic wounds if full-thickness grafts are required for deeper burns.

Given the heterogeneity in the design of available skin substitutes in terms of materials, cellularity, degradability, and the intended skin component for replacement, this systematic review exclusively focuses on permanent acellular dermal substitutes, as these form the greatest number of commercially available skin substitutes and will enable a more robust meta-analysis via the pooling of data. Acellular dermal substitutes typically utilize allografts or xenografts that have been decellularized and sterilized but retain cytokines and growth factors to encourage healing and integration of the patient’s own fibroblasts and endothelial cells into the matrix [3]. This encourages the formation of a neodermis by acting as a biosynthetic scaffold for fibroblast migration and re-vascularization whilst ensuring biocompatibility and reducing the immune response to the substitute [3]. The porous dermal matrix, which can be made from collagen, elastin, proteoglycans, or fully synthetic molecules, is typically covered by a non-biodegradable sealing membrane to limit moisture loss and provide anti-microbial protection [4]. A STSG is later placed over the dermal substitute to provide the epidermal component of the skin barrier, either in a single- or two-step procedure, whereby the application of STSG is delayed until the patient’s tissue has integrated with the substitute and the sealing membrane is removed. Thinner autografts are required for this purpose than for normal STSGs, enabling quicker regrowth and re-harvesting at donor sites [6].

The varying composition of dermal substitutes requires different methods of application. For example, the porous ultrastructure of Matriderm^®^ (MedSkin Solutions Dr. Suwelack, Billerbeack, Germany) allows the early migration of fibroblasts and angiogenic growth factors via the matrix, enabling vascularization within five days and hence application with STSG in a single step [7]. Contrastingly, the glutaraldehyde cross-links seen in Integra^®^ must be broken before vascularization and integration of the substitute can occur; hence, the application of STSG is delayed by 2–4 weeks to enable adherence and survival of the graft [7]. It has been suggested that a two-stage substitute application may be more appropriate in contaminated wounds to avoid unnecessary loss of STSG should infection become apparent in the initial days after application [8]. The key features of acellular dermal substitutes investigated by studies within this review are detailed below in Table 1, although not inclusive of all available substitutes.

With the ongoing production of new substitutes and research into their safety and performance in the clinical setting, this systematic review aimed to evaluate the current research relating to the efficacy, scar quality, and complication rates of acellular dermal substitutes and determine whether these substitutes are a viable alternative to conventional acute burn care.

## 2. Materials and Methods

### 2.1. Search Strategy

This systematic review was prospectively registered with PROSPERO (CRD42023412675) and conducted in accordance with the Preferred Reporting Items for Systematic Reviews and Meta-Analyses (PRISMA) guidelines [12]. To identify eligible publications, a search of the databases PubMed, Web of Science, and Cochrane Central Register of Controlled Trials (CENTRAL) was conducted. Following initial scoping searches to establish optimal search terms, the final search was conducted in March 2023 by two authors (I.P. and Z.A.) with the following Boolean terms: burn AND (dermal substitute OR skin substitute OR dermal regeneration template OR dermal regenerative matrix OR artificial skin equivalent OR biologic dressing OR Integra^®^ OR Matriderm^®^ OR Biodegradable Temporizing Matrix). No restrictions were placed on language or publication date to reduce the risk of systematic bias [13].

### 2.2. Inclusion and Exclusion Criteria

Inclusion criteria for eligible studies were established prior to searching to ensure all studies aligned with the following PICO of this systematic review. All studies had to involve the application of an approved acellular dermal substitute, such as, but not limited to, Integra^®^, Alloderm^®^ (LifeCell Corporation, Branchburg, NJ, USA), Matriderm^®^, or NovoSorb^®^ BTM, as the primary method of coverage of deep partial- and full-thickness acute burns that required excision and grafting in any age group. Studies were excluded if the substitute used did not have current regulatory approval, was of the autologous or allogeneic cellular type, was solely an epidermal substitute, was used to treat superficial or superficial partial-thickness burns, or if the substitute was used in non-burn pathologies or secondary burn reconstruction. A comparison of the outcomes from the relevant dermal substitute to routine skin grafting was necessary for the inclusion of RCTs to enable meta-analysis, but not for observational studies, to allow a more comprehensive assessment of the literature. The studies had to report at least one of our primary or secondary outcomes.

It was initially intended to only include randomized controlled trials (RCTs) in this systematic review, given that they form the highest level of evidence. However, following initial searches, the scope was broadened to include non-randomized trials and observational studies, given the difficulty in conducting robust RCTs for these interventions and the number of landmark observational studies in the field. Final searches were further limited to human studies only and filtered to the following study types if enabled by the database: RCTs, non-randomized trials, retrospective or prospective observational studies (i.e., cohort or case–control studies), excluding case series, case reports, narrative reviews, systematic reviews, editorials, letters, and conference proceedings.

### 2.3. Data Extraction

The studies were exported to EndNote, and duplicates were removed automatically. The studies were then independently screened by two reviewers (I.P. and Z.A.) by title, abstract, and full-text analysis to identify eligible publications; any disagreements were discussed and resolved without the need for a third reviewer. The following data was then extracted: study characteristics (study type, clinical setting and location, dermal substitute used, and any comparators), population demographics (sample size, age, male/female ratio, burn characteristics, and percentage total body surface area (%TBSA)) and outcome measures. When raw data was not available from the studies, no assumptions were made, and in some cases, study authors were contacted for further clarification. The primary outcomes for this systematic review were graft take and incidence of infection, whilst secondary outcomes included scar quality, graft loss, and length of acute hospital stay.

### 2.4. Risk of Bias Assessment

The risk of bias at the study level was assessed using the ‘Cochrane Risk-of-bias tool for randomized trials’ (RoB2) [14] for eligible RCTs and via the ‘Risk Of Bias In Non-randomized Studies—of Exposures’ (ROBINS-E) tool for observational studies [15]. Two researchers (I.P. and Z.A.) independently conducted a risk of bias assessment for each study and discussed and resolved any discrepancies to produce the final assessment.

### 2.5. Statistical Analysis

Due to heterogeneity in design, outcomes, and length of follow-up in the included studies, a meta-analysis was not appropriate for all outcomes. Where three or more RCTs reported the same outcome at comparable time points with sufficient raw data for calculation, a meta-analysis was conducted via RevMan 4.0 using a random effects model, reporting odds ratios or mean difference. The heterogeneity in the studies was assessed using I^2^, Chi^2^, or Tau^2^ statistics. In addition, a narrative synthesis was conducted of all studies reporting the previously discussed primary and secondary outcomes.

## 3. Results

### 3.1. Study Selection

**Figure 1:**
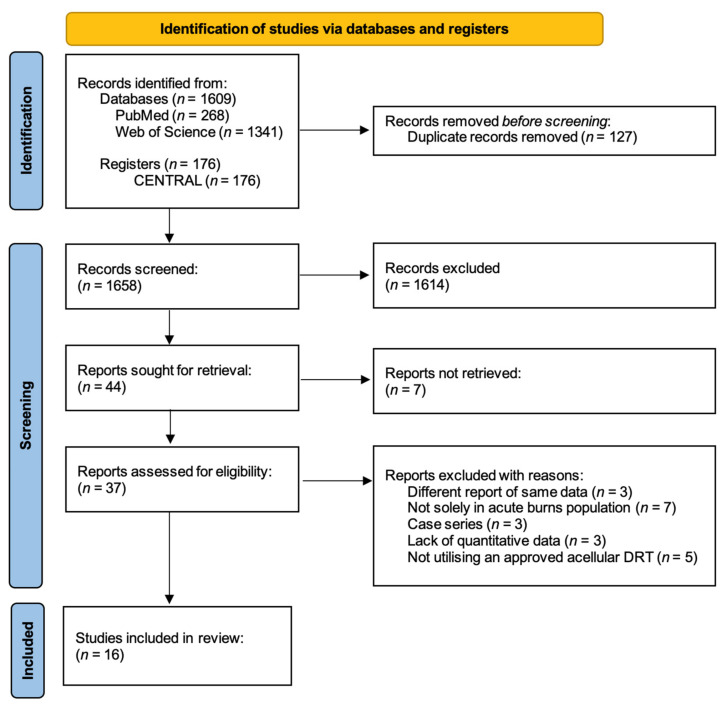
PRISMA flowchart for selection of studies.

Searches of PubMed, Web of Science databases, and CENTRAL register yielded 268, 1341, and 176 results, respectively. Following the removal of duplicates, 1658 studies were screened by title and then abstract, which yielded 44 studies for retrieval for full-text analysis. Seven of these reports were not retrieved due to a lack of published data for several clinical trials or an inability to access the full text. Of the remaining 37 studies, 16 were included in the final review following full-text assessment, consisting of 9 RCTs [16,17,18,19,20,21,22,23,24] and 7 observational studies [25,26,27,28,29,30,31]. A PRISMA flowchart [12] depicting this process is displayed in Figure 1.

### 3.2. Study Characteristics

The included studies spanned from 1988 to 2022 across a myriad of countries worldwide. Four studies were multi-center trials [16,18,27,28], and twelve were conducted in single burn departments. Studies were identified that used the acellular dermal substitutes Integra^®^, Matriderm^®^, and NovoSorb^®^ BTM. Only one study investigated NovoSorb^®^ BTM [28].

There was considerable variation in the age ranges of study samples: nine studies limited their sample to adults only, two to pediatrics, and five included patients of any age. There also was considerable diversity in the sample size of included studies, ranging from 10 to 270 patients, although it should be noted that most studies included multiple intervention sites from the same patients. All studies investigated dermal substitute use in deep partial- and full-thickness acute burns, which required excision and grafting. It should be noted that Busche et al. also included a separate group with superficial burns treated conservatively with dressings, which we will exclude from our analysis [26]. A total of 346 patients were included in the RCT analysis and 745 in the observational study analysis.

Table 2 and Table 3 summarize key study characteristics and outcomes, divided into RCTs and observational studies.

### 3.3. Primary Outcomes

#### 3.3.1. Take Rate

**Figure 2:**
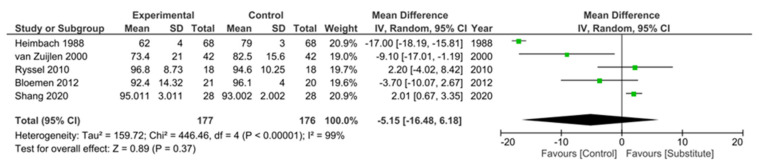
Forest plot to show results of meta-analysis for % graft take after substitute and control graft treatment [16,18,22,23,24].

Take rate was defined across the studies as the percentage of STSG that appears vascularized and adherent to the wound bed on which the dermal substitute was initially placed. Six RCTs [16,18,21,22,23,24] and three observational studies [27,28,29] examined the take rate. A meta-analysis of five RCTs with sufficient data was conducted, four of which used STSG [16,18,22,24] and one an intermediate thickness graft (ITSG) [23] as a control. Overall, the meta-analysis suggested that graft take was superior in the control group than with the substitute, but this did not reach significance (mean difference 5.15%, 95% CI [16.48, 6.18], *p* = 0.37) (Figure 2).

Both Heimbach et al. and van Zuijlen et al. [18,24], the largest of the included trials, demonstrated a significantly decreased graft take when a substitute was used (*p* < 0.0001 and *p* = 0.015, respectively). The remaining RCTs reported no significant difference in graft take [16,21,22], with the exception of Shang et al. [23], which significantly favored the substitute (*p* = 0.005), a surprising result given that their control method of ITSG typically offers superior outcomes to the more commonly used STSG [32].

Within the observational studies, a similar graft take to the later RCTs was reported, although without control interventions for comparison. Studies solely using Integra^®^ reported take rates of 87.7% (standard deviation not reported) and 85.5 ± 25.9, respectively [27,29], whilst the study from Lo et al. [28] experienced a take rate of 81.9 ± 18.8% with the use of NovoSorb^®^ BTM.

#### 3.3.2. Incidence of Infection

**Figure 3:**
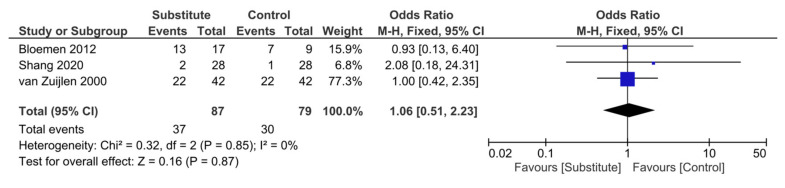
Forest plot to show results of meta-analysis of wound infection rates with substitute and control treatment [16,23,24].

Five RCTs [16,17,20,23,24] and four observational studies [25,27,28,30] included infection as a key outcome, either based on clinical assessment or microbiology results. Of these RCTs, three had sufficient raw data for a meta-analysis, which suggested that the odds of developing an infection when a substitute was used compared to conventional grafting were marginally higher but that this was not statistically significant (odds ratio 1.06, 95% CI [0.51–2.23], *p* = 0.87) (Figure 3).

The three RCTs included in this meta-analysis all individually reported no significant difference in infection rates between their substitute and control groups [16,23,24]. Concordantly, Branski et al. demonstrated no significant difference in wound infection rates or causative organisms between their Integra^®^ and STSG groups, with four patients in each experiencing invasive wound infections [17]. However, the RCT from Peck et al. was terminated early due to abnormally high infection rates in 85% of patients, which the researchers suggest may be due to the severity of burns in their sample (all >45% TBSA) or their lack of expertise in the use of Integra^®^ [20].

Of the observational studies, Heimbach et al. reported the lowest incidence of infection with its use of Integra^®^ in 216 patients (16.3%; 95% CI, 13.9–19.0%), with superficial and invasive infection occurring in 13.2% (95% CI, 11.0–15.7%) and 3.1% (95% CI, 2.0–4.5%), respectively [27]. Bargues et al. [25], who also used Integra^®^, reported much higher infection rates (42% overall, of which 71% were local and 29% were invasive infections), which they acknowledge may be due to their surgical technique, such as a longer delay in autograft placement compared to manufacturer recommendations (31.9 days versus 21 days). Lo et al. [28] reported an infection in 38.5% of patients treated with NovoSorb^®^ BTM. Phillips et al. [30] concluded there was no statistically significant difference in minor infection rates between Integra^®^ and Matriderm^®^ but that the incidence of major infection was significantly higher in their patients treated with Integra^®^ (*p* < 0.05).

### 3.4. Secondary Outcomes

#### 3.4.1. Scar Quality

Seven RCTs [16,17,19,21,22,23,24] and two observational studies [26,28] reported the resulting scar quality of the treated burn site. There was considerable variation in the time of follow up, ranging from three months to two years, and in the method used to assess scar quality, including the Vancouver Scar Scale (VSS), Patient and Observer Scar Assessment Scale (POSAS), Hamilton Burn Scar Score (HBSS) and Cutometer^®^ machine.

Three RCTs [16,19,24] reported no significant differences in scar quality. However, four reported a significant improvement with the use of dermal substitute versus conventional autograft: Branski et al. [17] using the HBSS at 12 and 18–24 months (*p* = 0.003 and *p* = 0.02, respectively), Shang et al. [23] with the VSS at multiple time points (*p* < 0.001 in all) and Ryssel et al. [22] using VSS at 6 months (*p* = 0.02). In their earlier study, Ryssel et al. [21] demonstrated improved scar elasticity when sheets of substitute and autograft were compared (*p* = 0.04) but not when meshed (*p* = 0.15).

Lo et al. [28] reported a significant improvement in the scar quality of the recipient site treated with NovoSorb^®^ BTM over time, with VSS measurements at three, six, and twelve months (*p* < 0.001). However, without a comparator, we are unable to ascertain whether this was improved by the substitute or simply followed the natural time course of scar maturation. Busche et al. [26] assessed each scar using three different tools at two years post-injury, as scar evaluation was the focus of their study. They reported no significant difference between scars from deep partial- and full-thickness burns treated with Matriderm^®^ or STSG, measured with the VSS or patient aspect of the POSAS, but significantly worse scores with the substitute in the observer scale of POSAS. An assessment of skin elasticity in Matriderm^®^-treated wounds with the Cutometer^®^ showed no statistically significant difference to normal skin, in contrast to the significant differences noted between normal skin and sites treated with STSG, indicating objectively superior elastic qualities in the burn scars treated with Matriderm^®^ [33].

#### 3.4.2. Graft Loss

Only three RCTs [16,19,20] and one observational study [30] reported the incidence of graft loss in their sample. Bloemen et al. [16] recorded graft loss (defined as failure of 5–100% of the graft) of 23% in the substitute group and 11% in the control STSG group, with an additional 9% of those in the substitute group also experiencing graft loss due to hematoma. However, there was no overall significant difference in the number of patients with these complications (*p* = 0.303). Lagus et al. [19] encountered a single patient who experienced graft loss due to a technical error. Contrastingly, 71% of the patients in the study from Peck et al. [20] experienced graft loss, although this can be attributed to the high rates of infection in this study and their protocol of removing the substitute within 0–3 days if there were signs of infection. Phillips et al. [30] reported that complete autograft loss was significantly higher in burns treated with Integra^®^ than Matriderm^®^ (*p* = 0.01).

#### 3.4.3. Length of Stay

The length of patients’ initial hospital admission for their acute burn was recorded in two RCTs [17,20] and three observational studies [25,30,31]. Results were varied, given the heterogeneity between study sample ages, %TBSA, routine burn practices, and complication rates.

The RCT by Branski et al. concluded there was no significant difference in length of stay between groups treated with Integra compared to those treated to split-thickness autografts or allografts (41 ± 4 days versus 39 ± 4 days, *p* = 0.49) [17]. Peck et al. [20] reported considerably longer stays than other studies, which may be explained by the severity of burns and high rates of infection in their sample. However, given their intra-individual paired study design, we could not determine the difference between those treated with the substitute or control autograft [20].

Within the observational studies, significantly shorter hospital admissions were observed when patients were treated with STSG compared to Integra^®^ in the study by Ryan et al. [31] (*p* < 0.001). However, when adjusted for severity risk factors, this did not reach significance, as the Integra population had a significantly higher mean TBSA burn and a greater incidence of inhalation injury (*p* < 0.001 and *p* = 0.04, respectively). Bargues et al. [25] reported more extensive hospital stays, with a mean of 96 ± 46 days, although without a comparator, we are unable to ascertain whether this is solely due to Integra^®^ use or may instead be attributed to the severity of their patients’ burns and high infection rates in the unit. Phillips et al. [30] reported a statistically significant shorter length of stay when treated with Matriderm^®^ over Integra^®^ (*p* < 0.05), although not significant when adjusted for %TBSA of the burn (*p* > 0.05).

### 3.5. Risk of Bias Assessment

**Figure 4:**
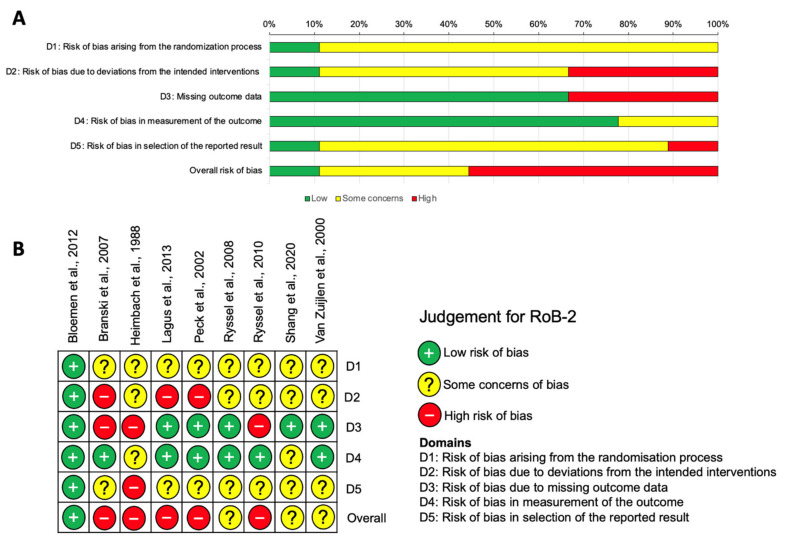
Risk of bias assessment in RCTs using the RoB2 tool [14]. (**A**) Summary chart of all studies assessed against each domain. (**B**) Risk of bias in individual studies across the 5 domains (D1–D5) [16,17,18,19,20,21,22,23,24].

Using the RoB2 tool for RCTs [14], we found that 55.56% of the articles were judged as having a high risk of bias [17,18,19,20,22], with another third of the articles judged as having ‘some concerns’ [21,23,24]. Only one study was graded as being at a low risk of bias [16]. Domains with a high risk of bias included risk of bias due to deviations from the intended interventions, where 33.33% of the articles were deemed to have a high risk of bias, with a further 55.56% judged as having some concerns. Missing outcome data was also a domain judged to have a high risk of bias in a third of the studies. These outcomes are depicted in Figure 4 at both the study (4A) and individual (4B) levels.

**Figure 5:**
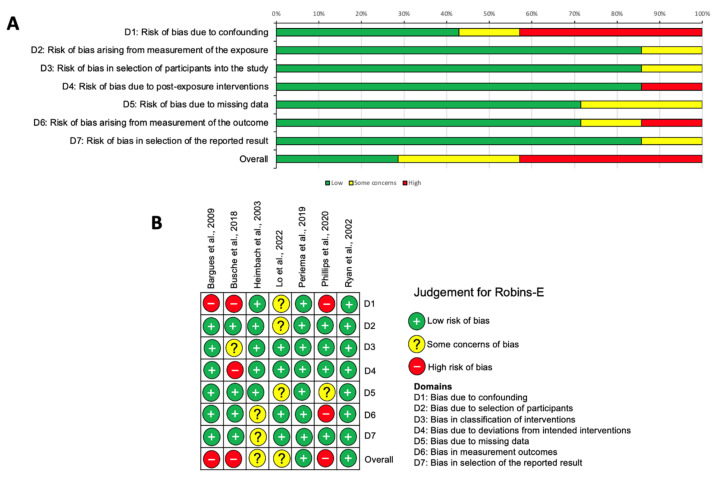
Risk of bias in observational studies using the ROBINS-E tool [15]. (**A**) Summary chart for risk of bias in all studies. (**B**) Individual risk of bias assessment in observational studies [25,26,27,28,29,30,31].

Across the included observational studies, appraised with the Robins-E tool [15], 42.9% of studies were judged as having a high risk of bias [25,26,30], with ‘some concerns’ identified overall in a further two studies [27,28]. Two studies were assessed to be at low risk of bias [29,31]. The highest risk of bias was observed in domain one (the risk of bias due to confounding), with other concerns identified with post-exposure interventions (domain four) and outcome measurements (domain six) [15]. These outcomes are depicted in Figure 5 at both the study (5A) and individual (5B) levels.

## 4. Discussion

This systematic review of acellular dermal substitutes provides an overview of studies investigating the safety and efficacy of this method of acute burn coverage. Based on our review criteria, studies were included that investigated three approved acellular dermal substitutes, although we note that several similar products are currently undergoing further trials. Our meta-analysis of studies demonstrated that whilst graft take was superior in the control group than in the substitute group, there was no significant difference in graft take between the use of dermal substitutes and our current gold-standard practices for acute burns, with observational studies also reporting comparable take rates. However, it should be noted that our meta-analysis displayed substantial heterogeneity (I^2^ = 99%), and included studies were judged to possess a high risk of bias, so conclusions should be treated with some caution. Of the included RCTs, the only two studies that reported a significantly worse graft take with the substitute were also the earliest studies [18,24], so it is likely that surgeons’ experience and expertise with substitutes have since increased, which may account for improved graft take in later research. Heimbach et al. [18], in fact, noted that centers that contributed more patients to their multi-center trial had improved take rates (*p* < 0.03) and suggested that a learning curve with the substitute application may be responsible for this result. Additionally, across all studies, with the exception of Peck et al. [20], there was minimal graft loss reported, which was generally attributed to technical error or infection of the wound site [16,19].

Our meta-analysis investigating the incidence of infection in acute burns patients also found no significant difference in those treated with dermal substitutes versus conventional care, although infection rates were marginally higher in the substitute group. However, infection rates remained high among the included observational studies. Given the lack of a control group for comparison among these observational studies, it is difficult to say whether these infection rates are a product of the substitute itself or the study settings, and the design of further robust comparative studies is recommended. There was limited data available assessing burn wound infection rates following conventional treatment, specifically in our included population, although a retrospective study assessing infection rates in patients with ≥20% TBSA burns treated with excision and grafting demonstrated infection rates of 39% [34]. This value is predominately in keeping with infection rates identified among dermal substitutes in all included studies, with the exception of those by Peck et al. and Bargues et al., who provide potential justification for their high infection rates [20,25]. Infection rates were predictably higher in studies including populations with larger burns [20,25,28], which is likely characteristic of the widespread systemic inflammatory response observed in burns over 20% TBSA [35]. Although outside the scope of this review, the studies that evaluated the use of negative pressure wound therapy (NPWT) in conjunction with dermal substitutes suggest this dual method can further reduce the risk of infection [16,29], and we recommend that future studies explore this area further.

Overall, our narrative synthesis of studies that reported scar quality suggests that the use of dermal substitutes offers a subjective and objective scar quality that is comparable, if not superior, to routine acute burn practices such as STSG. With the notable exception of Shang et al. [23], in which both the substitute and control scar quality worsened with time, most studies that assessed scar quality at multiple time points also demonstrated that scar quality of wounds treated with substitute improved over time as the scar matures, although this was not always significant compared to normal maturation. Whilst there was considerable heterogeneity in tools used to assess scar quality, the data suggest that the scar scales used all offer reasonable reliability and validity, although each is not without its flaws, whilst the Cutometer^©^ offers a reliable, objective method of burn scar assessment [36,37].

From the evidence available, we cannot conclusively determine whether the use of dermal substitutes affects the length of acute hospital stay, given the differences in routine burn care in individual burn units, regardless of whether the patient received the substitute or comparator. Overall, the five included studies, including length of stay as an outcome [17,20,25,30,31], reported hospital admission times in patients treated with dermal substitutes that are reflective of the predicted length of stay for the respective mean burn surface areas of their sample [38]. Whilst we cannot conclude whether these substitutes offer a significant difference due to contrasting results and a lack of comparator in some studies, there is currently no evidence to suggest that the use of dermal substitutes in acute burn care drastically increases the length of stay, and hence they remain a suitable adjunct to traditional grafting and dressings from this perspective.

We were initially keen to compare dermal substitutes to determine which may offer superior outcomes. Outcomes for acute deep burns may vary when treated with a matrix that requires a two-stage application with 2–4 weeks delay, compared to a dermal substitute applied with STSG in a single procedure, such as Matriderm^®^, due to differing times for the vascularization and integration of the substitute [7]. Therefore, results from our comparisons between Matriderm^®^ and other substitutes may need to be considered with caution as studies may have assessed certain outcomes, such as take rate, at different time points depending on the expected time for matrix integration. However, only one observational study compared these interventions, and no RCTs. Phillips et al. [30] retrospectively compared outcomes using Integra^®^ in a two-step and Matriderm^®^ in a one-step procedure in primary acute burn surgery. Whilst there was no significant difference between substitutes in most efficacy and safety outcomes, a significantly greater number of major infections (*p* < 0.05) and contractures (*p* < 0.005) were observed in Integra^®^ patients, although this population had significantly larger burns and higher rates of inhalational injury, both identified as risk factors for infection [39]. The Matriderm^®^ dermal matrix contains elastin, which is thought to improve extracellular matrix remodeling [40] and thus may account for this reduced wound contracture and would also explain the objectively superior scar elasticity demonstrated by Busche et al. when Matriderm^®^ was used compared to STSG treatment [26]. Given the wide variety of substitutes available on the market, RCTs comparing dermal substitutes in patients with similar demographics and similar injury profiles would be of value to determine which, if any, offer optimal efficacy and safety or whether the choice of substitute should remain at the discretion of the operating surgeon.

### Limitations

This review evaluated the safety and efficacy of acellular dermal substitutes in acute burn injury. However, there were several limitations in both the evidence included in our synthesis and our review processes that must be acknowledged. Whilst this review used RCTs, the highest level of evidence, to form the basis of our meta-analyses, we also included other observational study types in our narrative synthesis, which must rely on the quality and accuracy of previously recorded data to draw conclusions. Additionally, there was heterogeneity within the studies included in our meta-analysis due to differences in study design, the substitute and control used, and the number of stages in which the substitute was applied, although we attempted to limit this by only investigating acellular dermal substitutes. Some RCTs also had small sample sizes [19,20,21], which may have influenced results, especially given that the efficacy of the substitute is often highly dependent on the surgeon’s experience with this technique. A high risk of bias was detected in around half of the included studies. This may be due to the difficulty in producing blinded RCTs in this treatment area, due to the differing appearance and care required between the substitute and control, and also in the number of confounding factors associated with outcomes in burns.

## 5. Conclusions

This systematic review demonstrates that the use of acellular dermal substitutes for acute burns results in comparable efficacy and complication rates to STSGs and other conventional treatments and may produce improved scar quality. These substitutes therefore form an essential component in the burn surgeons’ toolkit, especially when donor sites are limited, to stage the closure of acute severe burns. However, given the high risk of bias in the included RCTs and observational studies, further rigorous research is necessary to draw more robust conclusions. We also encourage future exploration into the efficacy of NPWT in conjunction with substitutes and comparison between dermal substitutes to determine which, if any, offer superior outcomes in acute burns. As future studies on newer, fully synthetic substitutes emerge, the landscape may change once more, so we encourage clinicians to remain aware of ongoing research and adapt their practice accordingly.

## Figures and Tables

**Table 1:** Characteristics of included acellular dermal substitutes.

	Integra^®^ Dermal Regeneration Template [9]	Matriderm^®^ [10]	NovoSorb^®^ Biodegradable Temporizing Matrix (BTM) [11]
Manufacturer	Integra Life Science Corporation, Plainsboro, NJ, USA	MedSkin Solutions Dr. Suwelack AG, Billerbeck, Germany	PolyNovo^®^, Melbourne, Australia
First regulatory approval	1996	2005	2019
Number of layers	Bilaminar	Single layer	Bilaminar
Composition	Outer silicone layer, inner matrix of bovine tendon collagen, and shark chondroitin-6-sulfate	Type I, III, and V collagen and elastin scaffold	Outer sealing membrane bonded to porous matrix, both of synthetic polyurethane
Application procedure	2 stages: attached to debrided burn, then top silicone layer replaced with STSG 2–4 weeks later	Single stage: Matriderm^®^ and overlying STSG are placed on debrided wound simultaneously due to lack of upper epidermal layer in the template.	2 stages: attached to debrided wound, then outer layer replaced with STSG once capillary refill is observed in ~2–4 weeks

**Table 2:** Characteristics of included RCTs.

Study	Location	Sample Size	Substitute (Number of Sites)	Control Used (Number of Sites)	Target Population	Mean Age in Years, ±Standard Deviation (If Available)	Male: Female	Burn Characteristics	Mean %TBSA Burn, ±Standard Deviation If Available	Outcome Measures
Bloemen et al. (2012) [16]	The Netherlands ^1^	86	4 arms: Matriderm^®^, STSG and topical negative pressure therapy = DS-TNP (21), Matriderm^®^ and STSG = DS (23); STSG and TNP (22); STSG alone (20)		Adult	44 ± 17 (DS-TNP), 48 ± 19.4 (DS), 49 ± 13.3 (STSG and TNP), 53 ± 18.3 (STSG alone)	49:37	Deep dermal or full-thickness burns	Deep dermal or full-thickness burns needing skin transplant, TBSA ≤15%	Take rate, incidence of infection, pain scores, graft loss, occurrence of hematoma, need for regrafting, scar quality, and scar elasticity.
Branski et al. (2007) [17]	USA	20	Integra^®^ (10)	STSG (10)	Pediatric	7.4 (Integra^®^) and 6.2 (Control)	4:1	Burn size ≥50% TBSA and ≥40% TBSA full-thickness burn	Integra^®^ = 70 ± 5%, control = 74 ± 4%	Body composition, serum proteins, sepsis, wound infection, scar quality, and length of stay.
Heimbach et al. (1988) [18]	USA ^1^	106	Integra^®^ (68)	STSG (68)	Adult and pediatric	No data	3:1	Life-threatening deep partial- and full-thickness burns	46% ± 19%	Graft take, donor site morbidity, time to wound closure, surgeons’ assessment of substitute, mortality, and long-term assessment.
Lagus et al. (2013) [19]	Finland	10	Integra^®^ (10)	STSG (10) and Cellonex^®^ cellulose sponge (10)	Adult	36.8 ^2^	9:1	Full-thickness burn >20% TBSA on anterior side of body	35.8 ± 7.17	Mortality, scar quality, and histological analysis.
Peck et al. (2002) [20]	USA	9	Integra^®^ (9)	STSG allograft and Biobrane^®^ (9)	Adult and pediatrics	35.2 ^2^	No data	Deep partial- or full-thickness burns totaling >45% TBSA due to thermal injury	66.1 ± 13.86	Mortality, graft loss, wound or systemic infection, and length of stay.
Ryssel et al. (2008) [21]	Germany	10	Matriderm^®^ (28)	STSG (28)	Adult	49.5 ± 16.2	7:3	Full-thickness burns	45.6 ± 14.5	Substitute/graft take rate, need to regraft, and scar quality (using VSS).
Ryssel et al. (2010) [22]	Germany	18	Matriderm^®^ (18)	STSG (18)	Adult	45.1 ± 17.4	13:5	Full-thickness acute burns on the dorsum of both hands	43.3 ± 11.8	Substitute/graft take rate, need to regraft, scar quality (via VSS), range of motion (Finger-Tip-Palmar Crease-Distance (FPD) and Finger-Nail-Table-Distance (FNTD)).
Shang et al. (2020) [23]	China	56	Unspecified artificial dermis (28)	Intermediate thickness skin graft (ITSG) (28)	Adult	36.48 ± 3.47 (artificial dermis), 36.38 ± 3.51 (control)	31:25 ^2^	Total burn area >85% TBSA with deep partial-thickness burn area >50% TBSA and scar area >50% TBSA	No data	Healing time, scar quality (via VSS), graft take, infection rates, psychological status (self-rating anxiety and depression scales), and active recovery of function.
van Zuijlen et al. (2000) [24]	Netherlands	31	Matriderm^®^ (42)	STSG (42)	Adult	32.9 ± 19.3	18:13	Deep partial- and full-thickness acute burns	19.8 ± 14.5	Take rate, infection, need for reconstruction, and scar quality.

Notes: ^1^ multicenter trial; ^2^ calculated from existing data presented.

**Table 3:** Characteristics of included observational studies.

Study	Location	Study Design	Sample Size	Substitute (Number of Sites)	Control Used (Number of Sites)	Target Population	Mean Age in Years, ±Standard Deviation If Available	Male: Female	Burn Characteristics	Mean %TBSA Burn ±Standard Deviation If Available	Main Outcomes
Bargues et al. (2009) [25]	France	Retrospective	50	Integra^®^ (71)	None	Adult and pediatric	40 ± 15	35:15	Deep partial-thickness acute burns	45 ± 21	Incidence of infection, microbiology of infections, and length of stay.
Busche et al. (2018) [26]	Germany	Prospective	45	Matriderm^®^ (6)	STSG (49)	Adult	45	65:35	Scars from deep partial- and full-thickness burns treated acutely >2 years previously	No data	Burn scar evaluation with Cutometer, POSAS, and VSS.
Heimbach et al. (2003) [27]	USA ^1^	Prospective	216	Integra^®^ (841)	None	Adult and pediatric	34.74 ± 23.85	70:30	Life-threatening deep partial- to full-thickness burn	36.5 ± 24.7	Incidence of Integra infection, mortality, Integra, and autograft take rate.
Lo et al. (2022) [28]	Australia and France ^1^	Prospective	26	NovoSorb^®^ BTM (100)	None	Adult	45.2 ± 16.5	22:4	Deep partial- or full-thickness thermal burn 10–70% TBSA	47.5 ± 14.2	Substitute and STSG take, incidence of infection, adverse events, and scar quality (VSS at 12 months).
Pereima et al. (2019) [29]	Brazil	Retrospective	44	Integra^®^ (22)	Integra^®^ and negative pressure wound therapy (22)	Pediatric	No data	25:19	Deep partial- and full-thickness burns	No data	DRT and STSG take rates and time to maturation.
Phillips et al. (2020) [30]	UK	Retrospective	94	Matriderm^®^ (35) and Integra^®^ (59)		Adult and pediatric	28 ± 20.3 (Matriderm), 17 ± 17.7 (Integra)	No data	Deep partial- and full-thickness burns	Integra = 36.8 ± 23.3, Matriderm = 15.7 ± 20.9	DRT take, time to healing, complication rates, and graft loss.
Ryan et al. (2002) [31]	US	Retrospective	270	Integra^®^ (43)	STSG (227)	Adult	50 ± 21 (Integra), 46 ± 20 (control)	No data	Full-thickness burn ≥20%	Integra = 55 ± 19, Control = 59 ± 21	Mortality, LOS, and time to closure.

Notes: ^1^ multicenter trial.

## Data Availability

The data presented in this study can be found within the article.
